# Correlations of Molecular Weights of β-Glucans from Qingke (Tibetan Hulless Barley) to Their Multiple Bioactivities

**DOI:** 10.3390/molecules23071710

**Published:** 2018-07-13

**Authors:** Shang Lin, Huan Guo, Min Lu, Ming-Yuan Lu, Jia Duo Bu Gong, Lu Wang, Qing Zhang, Wen Qin, Ding-Tao Wu

**Affiliations:** 1 Institute of Food Processing and Safety, College of Food Science, Sichuan Agricultural University, Ya’an 625014, China; slsicau@163.com (S.L.); ghscny@163.com (H.G.); mlsicau@163.com (M.L.); MY_Lusicau@163.com (M.-Y.L.); zhangqing@sicau.edu.cn (Q.Z.); 2 Tibetan Gan-Yu-Cang Agricultural Products Development Co., Ltd., Changdu 855700, China; gonggyc@126.com (J.D.B.G.); wanglugyc@126.com (L.W.)

**Keywords:** Qingke, β-glucans, molecular weight, binding properties, inhibitory activities on digestive enzymes, anti-inflammatory activity, anti-cancer activity

## Abstract

β-glucans have been considered the major bioactive components in Qingke (Tibetan hulless barley). However, the structure–function relationships of β-glucans from Qingke have seldom been investigated. Whether the bioactivities of Qingke β-glucans are closely correlated to their molecular weights remains unknown. Therefore, in order to explore Qingke β-glucans as functional/healthy food ingredients for industrial applications, and to better understand their structure–function relationships, correlations of molecular weights of Qingke β-glucans to their in vitro binding properties, inhibitory activities on digestive enzymes (α-amylase and pancreatic lipase), anti-inflammatory activities, and anticancer activities were systematically investigated. Results showed that the in vitro binding properties and the inhibitory activities on α-amylase and pancreatic lipase of Qingke β-glucans were positively correlated to their molecular weights. However, the anti-inflammatory activities of Qingke β-glucans increased as their molecular weights decreased. Furthermore, Qingke β-glucans exhibited selectively anti-cancer activities in vitro. Positive and negative correlations of molecular weights to inhibitory effects against A549 cells and MDA-MB-231 cells were observed, respectively. However, the inhibitory effects of Qingke β-glucans against HCT116 cells were not associated with their molecular weights. Results suggested that the molecular weights of Qingke β-glucans significantly affected their bioactivities, which was beneficial for a better understanding of their structure–function relationships. Moreover, results showed that Qingke β-glucans could be further explored as functional/healthy food ingredients for industrial applications due to their multiple health benefits.

## 1. Introduction

Qingke (Tibetan hulless barley, *Hordeum vulgare* L.), a cultivar of hulless barley that grows at high altitude, is the staple food for Tibetans, and an important economic and industrial crop in the Tibetan Plateau [1,2]. Over the past decade, due to its health benefits, increasing interest in Qingke as a functional/healthy food has been noticed. Epidemiological studies have associated the regular consumption of the whole barley flour with its potential to reduce the risk of certain diseases, such as hyperlipidemia, diabetes, colonic cancer, high blood pressure, and gallstones [3]. Generally, β-glucans, composed of d-glucopyranose units linked through (1 → 4) and (1 → 3) glycosidic bonds, are considered the major bioactive components in barley [4,5,6], which have various bioactivities, such as antioxidant [7], anti-inflammatory [8,9], anti-cancer [10,11], immunomodulatory [12], cardioprotective [13], anti-diabetic [7,14], and anti-obesity effects [15]. Indeed, β-glucans are regarded as important functional ingredients to lower plasma cholesterol, reduce glycemic response, and promote weight management [5].

Generally, the molecular weights of β-glucans are strictly associated with their biological activities [8]. Several studies have shown that the decrease in molecular weights of β-glucans from oat enhances their antioxidant activities [8,11,16,17]. Furthermore, the immune-enhancing activity [18], anti-diabetic activity [14], anti-proliferative activity [17], anti-inflammatory activity [19], and in vitro bile acid-binding capacity [20] of β-glucans from oat are enhanced with the decrease of their molecular weights, which suggests that molecular weight plays an important role in their physiological effects. However, the bioactivities and structure–function relationships of β-glucans from Qingke have seldom been investigated. Whether the biological activities of Qingke β-glucans are closely correlated to their molecular weights remains unknown. Therefore, in order to explore Qingke β-glucans as functional/healthy food ingredients for industrial applications, and to better understand the structure–function relationships of Qingke β-glucans, correlations of molecular weights of Qingke β-glucans to their in vitro binding properties, inhibitory activities on digestive enzymes (α-amylase and pancreatic lipase), anti-inflammatory activity, and anti-cancer activity were systematically investigated in the present study.

## 2. Results and Discussions

### 2.1. Characterization of Qingke β-Glucans with Different Molecular Weights

**Figure 1:**
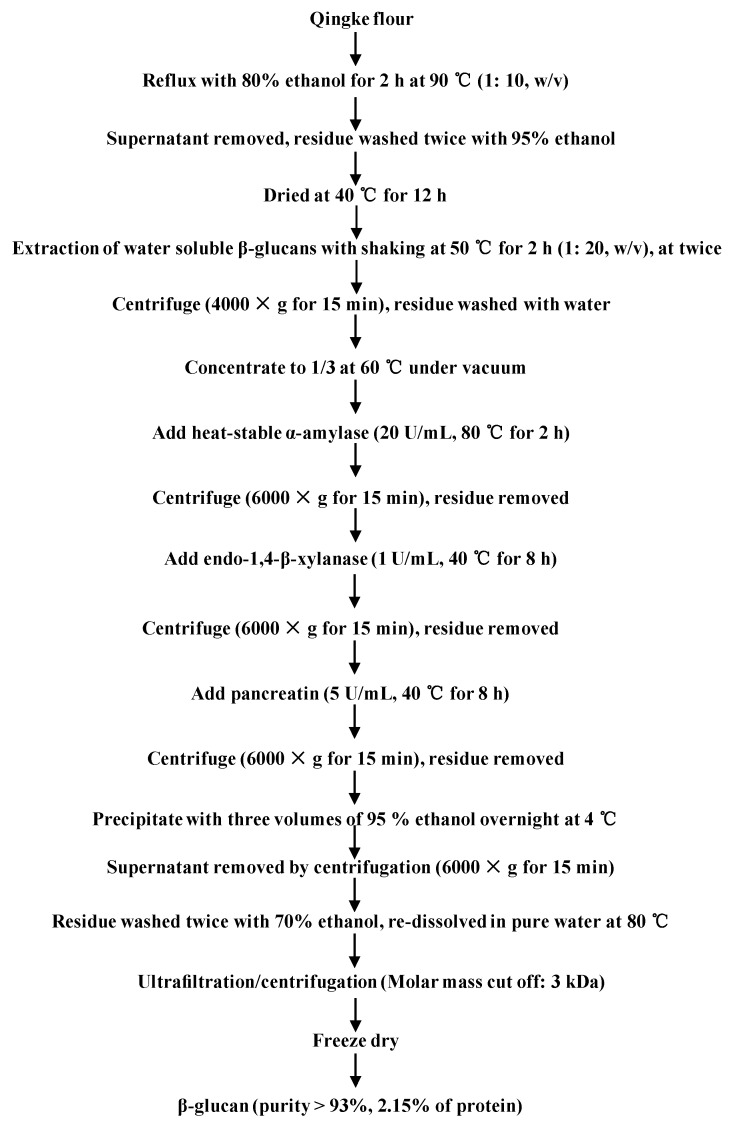
Flow diagram of extraction and purification of Qingke β-glucans.

**Figure 2:**
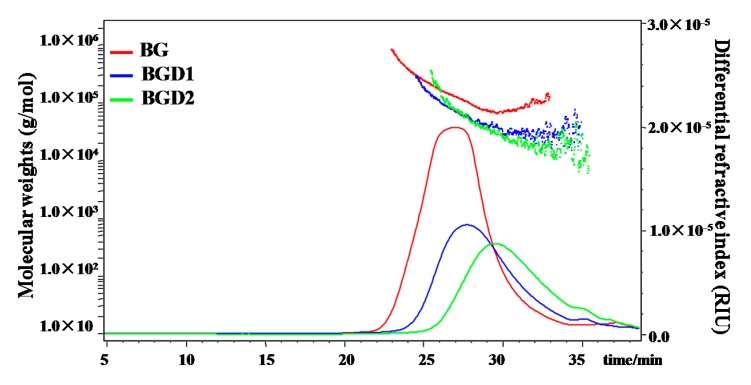
HPSEC-RID chromatograms and molecular weights of Qingke β-glucans. **BG**, Qingke β-glucan; **BGD1**, Qingke β-glucan with acid hydrolysis for 10 min; **BGD2**, Qingke β-glucan with acid hydrolysis for 20 min.

Qingke β-glucans with high molecular weight (BG) were partially hydrolyzed by trifluoroacetic acid for 10 min to obtain β-glucans with medium molecular weight (BGD1), and for 20 min to obtain β-glucans with low molecular weight (BGD2), respectively. As shown in Table 1, results showed that the purity of BG was determined to be 93.8% by the mixed-linkage β-glucan assay kit (for the measurement of 1,3:1,4-β-d-glucan in cereal grains), which suggested that the proposed method (Figure 1) for the extraction and purification of β-glucans from Qingke flour was efficient. Furthermore, results showed that the purities of BGD1 and BGD2 were determined to be 93.1% and 93.3%, respectively, by the mixed-linkage β-glucan assay kit, which suggested that the chemical compositions of BG, BGD1, and BGD2 were similar. Indeed, the contents of proteins in BG, BGD1, and BGD2 were 2.15%, 0.72%, and 0.49%, respectively, which could be neglected in further bioassays. Moreover, Figure 2 shows the HPSEC-RID chromatograms and molecular weight distributions of BG, BGD1, and BGD2. Results showed that the molecular weight of BG was 1.962 × 10^5^ (±0.54%) g/moL, which is in accordance with previous studies that showed that the molecular weights of water-soluble β-glucan in barley ranged from 1.0 × 10^5^ to 7.7 × 10^5^ g/moL [21,22]. The molecular weights varied by cultivar, contents, growing environment, and extraction conditions [22]. Additionally, the molecular weights of BGD1 and BGD2 were observed to be 0.638 × 10^5^ (±2.35%) g/moL and 0.358 × 10^5^ (±2.65%) g/moL, respectively. Furthermore, a minor peak around 35 min existed in BGD1 and BGD2. This might be due to small hydrolysates of BG after acid hydrolysis, which was not sufficiently removed by ultracentrifugation. Considering the low peak area, these small molecules had no significant effects on the molecular weights of BGD1 and BGD2. Therefore, the minor peak around 35 min in both BGD1 and BGD2 could be neglected in further bioassays. Moreover, the polydispersities of BG, BGD1, and BGD2 were determined to be 1.586, 1.40, and 1.46, respectively. Meanwhile, the intrinsic viscosities of BG, BGD1 and BGD2 were determined to be 1.86 dL/g, 0.98 dL/g, and 0.58 dL/g, respectively, which indicated that the intrinsic viscosities were positively correlated to the molecular weights [23].

### 2.2. Correlation of Molecular Weights of Qingke β-Glucans to Their Binding Properties

Previous studies have shown that the molecular weight of β-glucans plays an important role in their in vitro binding capacities. For instance, the bile acid-binding capacity of β-glucans from oat flour increases as the molecular weight decreases [20]. However, the bile acid-binding capacity, oil-binding capacity, and water-binding capacity of β-glucans from barley flour decrease as the molecular weight decreases [22]. The relationships between binding property and molecular weight vary according to the different sources of β-glucans. So, whether the binding properties of β-glucans from Qingke are positively correlated to their molecular weights remains unknown. In vitro fat-, cholesterol-, and bile acid-binding capacities of Qingke β-glucans with different molecular weights were summarized in Table 2. Results showed that the capacities of fat binding, cholesterol binding, and bile acid binding of BG, BGD1, and BGD2 ranged from 2.23 ± 0.03 to 1.39 ± 0.02 g/g, from 40.37 ± 0.43 to 10.56 ± 0.28 mg/g, and from (26.49 ± 0.18)% to (11.54 ± 0.25)%, respectively. Meanwhile, the significantly (*p* < 0.05) highest fat-binding capacity (2.23 ± 0.03 g/g), cholesterol-binding capacity (40.37 ± 0.43 mg/g), and bile acid-binding capacity (26.49% ± 0.18%) were observed in BG, followed by lower in BGD1, and the lowest in BGD2. Results showed that the fat-binding, cholesterol-binding, and bile acid-binding properties of Qingke β-glucans were positively correlated to their molecular weights (from 1.962 × 10^5^ to 0.358 × 10^5^ g/mol) and intrinsic viscosities (from 1.86 to 0.58 dL/g), which suggested that the binding properties of Qingke β-glucans depended on their high molecular weight fractions. Results were in accordance with previous studies that the binding capacities of β-glucans from barley decreased as their molecular weights (from 6.064 × 10^5^ to 0.554 × 10^5^ g/mol) and apparent viscosities decreased (from 11.2 cP to 5.4 cP, 2% of β-glucans) [22]. However, this positive correlation is opposite to that of β-glucans with high molecular weights (from 2.06 × 10^5^ to 9.50 × 10^5^ g/mol) from oats [20], which suggested that an appropriate range of molecular weights of β-glucans was required for preserving their high level of binding properties. Furthermore, compared with cellulose (a positive control), BG, BGD1, and BGD2 showed a significantly higher fat-binding capacity. Indeed, the cholesterol-binding capacities of BG and BGD1 were also higher than that of cellulose (a positive control). However, the bile acid-binding capacities of BG, BGD1, and BGD2 were lower than that of cholestyramine (a positive control), but significantly higher than that of cellulose (a negative control). Results suggested that Qingke β-glucans had the potential to be explored as functional/healthy food ingredients for the prevention of diseases such as hypercholesterolemia and hyperlipidemia.

### 2.3. Correlation of Molecular Weights of Qingke β-Glucans to Their Inhibitory Activities on α-Amylase and Pancreatic Lipase

**Figure 3:**
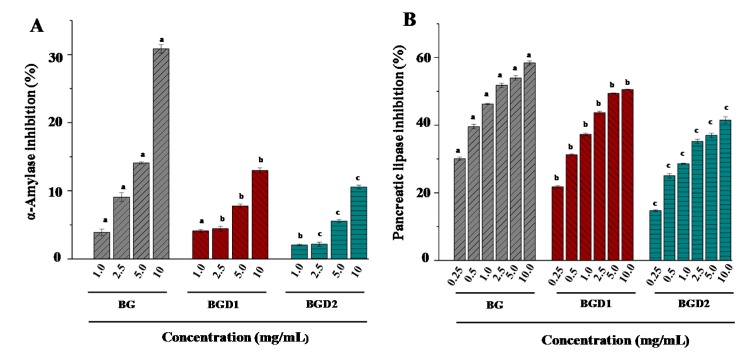
In vitro inhibitory activities of Qingke β-glucans on α-amylase (**A**) and pancreatic lipase (**B**). **BG**, Qingke β-glucan; **BGD1**, Qingke β-glucan with acid hydrolysis for 10 min; **BGD2**, Qingke β-glucan with acid hydrolysis for 20 min; the error bars are standard deviations; significant (*p* < 0.05) differences are shown by data bearing different letters (a–c); statistical significance tests were carried out by ANOVA.

Inhibition of fats and carbohydrates degrading enzymes can reduce obesity and type 2 diabetes [24]. Therefore, the in vitro inhibitory effects of Qingke β-glucans against key enzymes relevant for obesity (pancreatic lipase) and type 2 diabetes (α-amylase) were investigated. Figure 3 showed the inhibitory effects of Qingke β-glucans with different molecular weights on α-amylase and pancreatic lipase. Results showed that the inhibitory effects of Qingke β-glucans on α-amylase and pancreatic lipase significantly increased as the concentrations increased. Furthermore, the α-amylase inhibition effects of BG, BGD1, and BGD2 decreased as their molecular weights (from 1.962 × 10^5^ to 0.358 × 10^5^ g/mol) and intrinsic viscosities (from 1.86 to 0.58 dL/g) decreased (Figure 3A). The high viscosity of Qingke β-glucans might prevent α-amylase from coming into contact with the substrate (soluble starch), resulting in high α-amylase inhibition effects. Briefly, the highest α-amylase inhibition (30.85%) was observed in BG at a concentration of 10 mg/mL, followed by lower BGD1 (12.97%), and the lowest BGD2 (10.56%). Results suggested that the in vitro inhibitory effect of Qingke β-glucans on the α-amylase was positively correlated to their molecular weights and intrinsic viscosities. Results were in accordance with previous studies that showed that the anti-diabetic effect of β-glucans with high molecular weight from oat bran was greater than that of β-glucans with low and medium molecular weights [14]. Moreover, the pancreatic lipase inhibition effects of BG, BGD1, and BGD2 also decreased as their molecular weights and intrinsic viscosities decreased at concentrations of 0.25 to 10.0 mg/mL (Figure 3B). The high viscosity of Qingke β-glucans might also prevent the pancreatic lipase from coming into contact with the substrate. Indeed, the highest pancreatic lipase inhibition (58.44%) was also observed in BG at a concentration of 10 mg/mL, followed by lower BGD1 (50.55%), and the lowest BGD2 (41.48%), which suggested that the pancreatic lipase inhibition effects of Qingke β-glucans were positively correlated to their molecular weights and intrinsic viscosities. In addition, the IC_50_ values of pancreatic lipase inhibition of BG and BGD1 were measured as 2.947 mg/mL and 8.185 mg/mL, respectively, which further confirmed that the inhibitory effect of Qingke β-glucans on the pancreatic lipase decreased as their molecular weights decreased. Furthermore, compared with the positive control (orlistat, IC_50_ = 2.051 mg/mL), Qingke β-glucans exerted strong pancreatic lipase inhibition effects. Results suggest that the hypolipidemic effect of whole Qingke flour may be partially attributed to the strong pancreatic lipase inhibition effect of Qingke β-glucans [25].

### 2.4. Correlation of Molecular Weights of Qingke β-Glucans to Their In Vitro Anti-Inflammatory Activities

**Figure 4:**
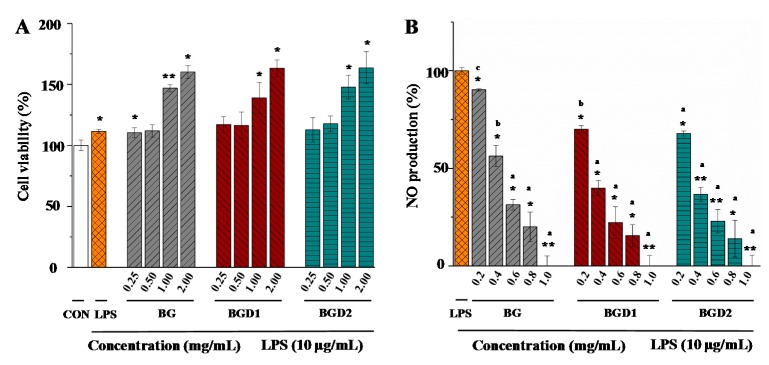
Cell viability (**A**) and NO production (**B**) of RAW264.7 macrophages treated with Qingke β-glucans. **BG**, Qingke β-glucan; **BGD1**, Qingke β-glucan with acid hydrolysis for 10 min; **BGD2**, Qingke β-glucan with acid hydrolysis for 20 min; The error bars are standard deviations; the differences of cell viability between sample and control are significant at * *p* < 0.05, ** *p* < 0.01; the differences of NO production between sample and LPS control group are significant at * *p* < 0.05, ** *p* < 0.01; significant (*p* < 0.05) differences of NO production among BG, BGD1 and BGD2 are shown by data bearing different letters (a–c).

Inflammation is an adaptive response that is triggered by foreign pathogens or tissue injury [26]. Macrophages play vital roles in initiation, maintenance, host defense, promotion, and resolution of inflammation. LPS-induced RAW264.7 macrophage is a well-established model for in vitro anti-inflammatory effect testing, which has been widely applied for the evaluation of anti-inflammatory activities of natural products [27]. NO is a major indicator of inflammation, and polysaccharides exhibiting NO inhibition may possess anti-inflammatory activity [28]. Therefore, in order to better understand the molecular weight-anti-inflammatory activity relationship of Qingke β-glucans, the effects of Qingke β-glucans with different molecular weights on NO production were investigated by using the LPS-induced RAW264.7 macrophage model. Different concentrations (from 0.25 mg/mL to 2.0 mg/mL) of Qingke β-glucans were investigated for cell viability using the MTT assay. As shown in Figure 4A, all concentrations of Qingke β-glucans had no cytotoxic effects on RAW264.7 macrophage. Once macrophages are activated in the presence of LPS, a naturally high content of pro-inflammatory molecules, such as NO, are produced. Figure 4B showed the inhibition of NO production in LPS-induced RAW264.7 macrophages. Results showed that a significant increase of NO production was induced by LPS. Compared with LPS-treated control cells, the levels of NO production significantly decreased in a concentration dependent manner in all Qingke β-glucans treated cells (Figure 4B). Results suggested that Qingke β-glucans exerted potential anti-inflammatory activities, which were in accordance with previous studies that showed that barley β-glucans with a molecular weight of about 1.4 × 10^5^ g/mol had significant anti-inflammatory activity [9]. Furthermore, the inhibitions of NO production induced by BG, BGD1, and BGD2 at the concentrations of 0.6 mg/mL to 1.0 mg/mL showed no significant differences. However, at a low concentration of 0.2 mg/mL, the NO production of BG, BGD1, and BGD2 differed significantly (*p* < 0.05). The lowest NO production was observed in BGD2 (67.81%), followed by higher BGD1 (70.01%), and the highest BG (90.26%). Results suggested that the anti-inflammatory activities of Qingke β-glucans increased as their molecular weights decreased from 1.962 × 10^5^ (±0.54%) g/mol to 0.358 × 10^5^ (±2.65%) g/mol, which was similar to the results of previous studies [19]. Indeed, the low intrinsic viscosity and high solubility of BGD2 might also contribute to its higher anti-inflammatory activity. BGD2 with a relatively low macular weight and high solubility might easily enter into the cells. The IC_50_ values of BG, BGD1, and BGD2 were determined to be 0.439 mg/mL, 0.314 mg/mL, and 0.301 mg/mL, respectively, which further confirmed that BGD2 (low molecular weight β-glucan) exerted the highest anti-inflammatory activity.

### 2.5. Correlation of Molecular Weights of Qingke β-Glucans to Their In Vitro Anti-Cancer Activities

**Figure 5:**
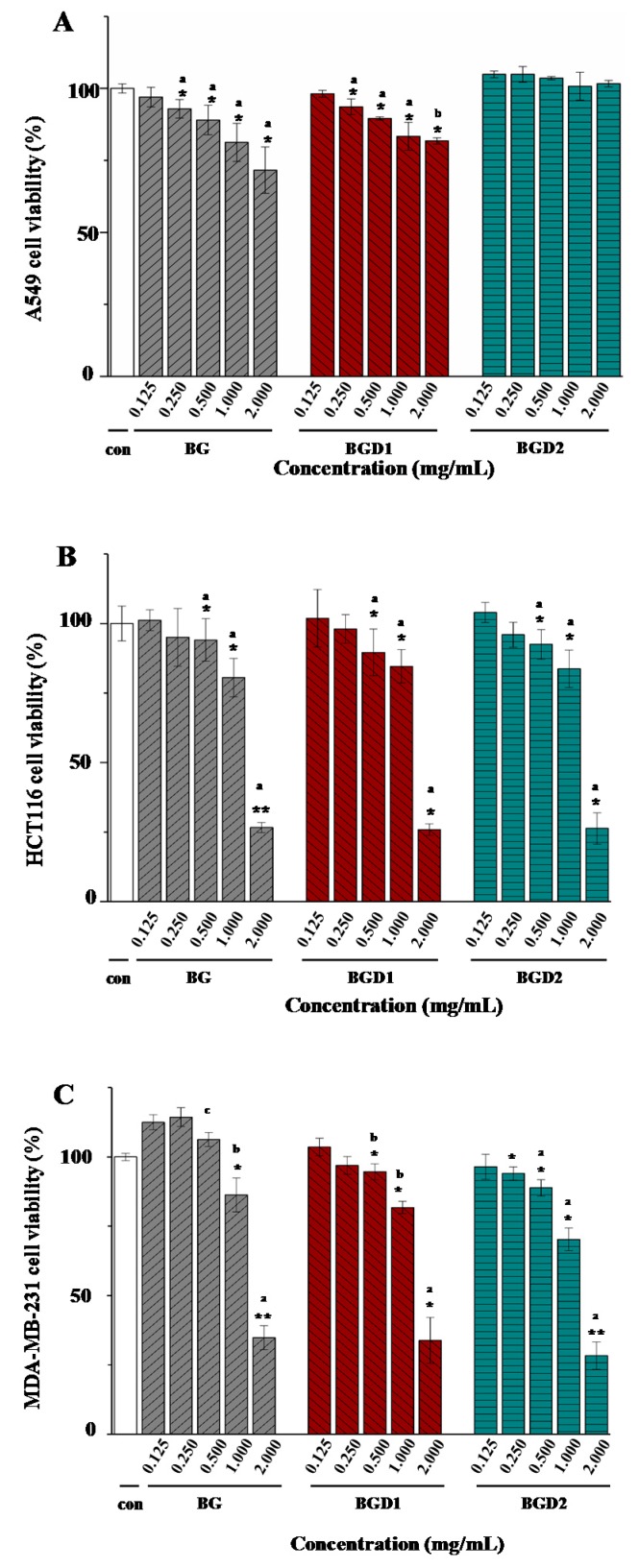
In vitro anti-cancer activities of Qingke β-glucans against A549 (**A**); HCT116 (**B**); and MDA-MB-231 (**C**) cells. **BG**, Qingke β-glucan; **BGD1**, Qingke β-glucan with acid hydrolysis for 10 min; **BGD2**, Qingke β-glucan with acid hydrolysis for 20 min; The error bars are standard deviations; the differences of cell viability between sample and control groups are significant at * *p* < 0.05, ** *p* < 0.01; significant (*p* < 0.05) differences of cell viability among BG, BGD1, and BGD2 are shown by data bearing different letters (a–c).

To assess the anti-cancer activity of Qingke β-glucans, the in vitro growth inhibitory effects of Qingke β-glucans against human lung cancer cells (A549), human colon cancer cells (HCT116), and human breast cancer cells (MDA-MB-231) were investigated. As shown in Figure 5, the in vitro growth inhibitory effects of Qingke β-glucans against A549 cells, HCT116 cells, and MDA-MB-231 cells differed significantly. Briefly, in the case of A549 cells, the in vitro growth inhibitory effects of Qingke β-glucans significantly decreased as their molecular weights decreased from 1.962 × 10^5^ (±0.54%) g/mol to 0.358 × 10^5^ (±2.65%) g/mol (Figure 5A). Indeed, at the concentration of 2.0 mg/mL, the highest inhibition rate against A549 cells was observed as 28.38% in BG, followed by lower BGD1 (18.25%), and no inhibition of BGD2 against A549 cells. Results showed that the in vitro growth inhibition effects of Qingke β-glucans against A549 cells were positively correlated to their molecular weights, which suggested that the high molecular weight of Qingke β-glucans was required for preservation of their inhibitory effects against A549 cells. Furthermore, the in vitro growth inhibitory effects of Qingke β-glucans against HCT116 cells are shown in Figure 5B. Results showed that Qingke β-glucans significantly (*p* < 0.05) inhibited the growth of HCT116 cells at concentrations from 0.5 mg/mL to 2.0 mg/mL. The IC_50_ values of BG, BGD1, and BGD2 were observed to be 1.479 mg/mL, 1.484 mg/mL, and 1.498 mg/mL, respectively, which indicated that Qingke β-glucans exerted strong inhibitory activity against HCT116 cells. Meanwhile, the in vitro growth inhibitory effects of BG, BGD1, and BGD2 against HCT116 cells showed no significant difference. Results showed that Qingke β-glucans with relatively low molecular weight also exerted strongly anti-cancer activity, which was similar to the results of previous studies [10]. Moreover, as shown in Figure 5C, Qingke β-glucans also significantly (*p* < 0.05) inhibited the growth of MDA-MB-231 cells at concentrations from 0.5 mg/mL to 2.0 mg/mL, and the IC_50_ values of BG, BGD1, and BGD2 were observed to be 1.681 mg/mL, 1.618 mg/mL, and 1.384 mg/mL respectively. Results indicated that the in vitro growth inhibitory effects of Qingke β-glucans against MDA-MB-231 cells significantly (*p* < 0.05) increased as their molecular weights decreased from 1.962 × 10^5^ (±0.54%) g/mol to 0.358 × 10^5^ (±2.65%) g/mol, which suggested that the in vitro growth inhibition effects of Qingke β-glucans against MDA-MB-231 cells were negatively correlated to their molecular weights. These results suggested that Qingke β-glucans exhibited selective anti-cancer activity in vitro, which was in accordance with previous studies that showed that oat and barley β-glucans possess potential anticancer activities in vitro [10,11]. Moreover, the negative correlation of molecular weights of Qingke β-glucans to their in vitro inhibitory effects against MDA-MB-231 cells was similar to previous studies where the in vitro anti-cancer activities of oat and barley β-glucans increased as their molecular weights decreased [11,17,29].

## 3. Materials and Methods

### 3.1. Material and Chemicals

Qingke (blue Qingke, *Dinqing*) was collected from Changdu (altitude > 4000 m), Tibet, China. Trifluoroacetic acid, sodium cholate, sodium deoxycholate, sodium glycocholate, sodium taurocholate, cholesterol, oleic acid, bovine serum albumin, cellulose, soluble starch, pancreatic lipase, α-amylase, *p*-nitrophenyl acetate, dimethyl sulfoxide (DMSO), streptomycin, 3-(4,5-dimethylthiazol-2-yl-)2,5-diphenyltetrazolium bromide (MTT), lipopolysaccharide (LPS), and Griess reagent were purchased from Sigma-Aldrich (St. Louis, MO, USA). Heat-stable α-amylase, pancreatin from porcine pancreas, and a free cholesterol assay kit were purchased from Solarbio (Beijing, China). The mixed-linkage β-glucan assay kit and endo-1,4-β-xylanase were obtained from Megazyme (Wicklow, Ireland). Orlistat and cholestyramine were purchased from a local pharmacy in Ya’an. All other reagents and chemicals used were of analytical grade.

### 3.2. Extraction and Purification of β-Glucan from Qingke

Qingke samples were dried at a temperature of 45 °C for two days, and then the samples were milled into whole Qingke flours. Subsequently, the whole Qingke flours were passed through a 60-mesh size screen. β-glucans from the whole Qingke flours were extracted and purified according to a previously reported method with some modifications [6]. Figure 1 shows the flow chart for extraction and purification of β-glucans from the whole Qingke flour. Briefly, 50 g of Qingke flours were refluxed with 500 mL of 80% (*v*/*v*) ethanol at 90 °C for 2 h. Then the water-soluble β-glucans were extracted twice with 1000 mL of pure water for 2 h at 50 °C. After centrifugation (4000× *g* for 15 min), the extracts were combined and concentrated to 1/3 of the original volume by a rotary evaporator (RE-52AA, Yarong Company, Shanghai, China) under a vacuum at 60 °C. Then, the heat-stable α-amylase (20 U/mL), endo-1,4-β-xylanase (1 U/mL), and pancreatin (5 U/mL) were sequentially used to remove starch, soluble arabinoxylans, and proteins, respectively. Subsequently, the extracts were precipitated with three volumes of 95% (*v*/*v*) ethanol overnight at 4 °C. The precipitations were washed twice with 70% of ethanol, and redissolved in pure water at 80 °C. After centrifugation, the supernatant was transferred to an Amicon ultracentrifugal filter device (molecular weight cutoff: 3 kDa, Millipore, Billerica, MA, USA). Then the compounds with molecular weight below 3 kDa were thoroughly removed by centrifugation (3500× *g* for 25 min) six times. Finally, the soluble β-glucans were freeze-dried. The yield of the soluble β-glucans (BG) was 1.6%, and the purity of BG was determined by a mixed-linkage β-glucan assay kit. The content of protein in BG was determined by the Bradford method.

### 3.3. Preparation of Qingke β-Glucans with Different Molecular Weights

Qingke β-glucans with different molecular weights were prepared by partial acid hydrolysis. Briefly, 600 mg of Qingke β-glucans with high molecular weight were dissolved in 120 mL of pure water, and then mixed with 120 mL of trifluoroacetic acid (1 M). Then the mixture was incubated at 90 °C for 10 min to obtain β-glucans with medium molecular weight (BGD1), or for 20 min to obtain β-glucans with low molecular weight (BGD2), respectively. The mixture was neutralized with 4 M sodium hydroxide. Subsequently, the mixture was transferred to an ultracentrifugal filter device (molecular weight cutoff: 3 kDa). Then the hydrolysates with the molecular weight below 3 kDa were thoroughly removed by centrifugation (4000× *g* for 25 min) for three times. Finally, BGD1 with medium molecular weight and BGD2 with low molecular weight were freeze-dried and stored at −20 °C for further analysis. Afterwards, BGD1 and BGD2 were obtained, and weighed as 332 mg (yield, 55.33%) and 281 mg (yield, 46.83%), respectively. Furthermore, the purities of BGD1 and BGD2 were determined by the mixed-linkage β-glucan assay kit.

### 3.4. Determination of Molecular Weights

The absolute molecular weights (*M_w_*) and polydispersities (*M_w_/M_n_*) of BG, BGD1, and BGD2 were measured by high-performance size-exclusion chromatography coupled with a multi-angle laser light scattering and refractive index detector (HPSEC-MALLS-RID) according to a previously reported method with minor modifications [30]. In brief, HPSEC-MALLS-RID measurements were carried out on a multi-angle laser light-scattering detector (MALLS, DAWN HELEOS, Wyatt Technology Co., Santa Barbara, CA, USA) with an Agilent 1260 series LC system (Agilent Technologies, Palo Alto, CA, USA). TSK-Gel G5000PWXL (300 mm × 7.8 mm, i.d.) and TSK-Gel G3000PWXL (300 mm × 7.8 mm, i.d.) were used in series at 30 °C. The MALLS instrument was equipped with a He-Ne laser (λ = 658 nm). An Optilab rEX refractometer (RID, DAWN EOS, Wyatt Technology Co., Santa Barbara, CA, USA) was simultaneously connected. The mobile phase was 0.9% NaCl aqueous solution at a flow rate of 0.5 mL/min. The sample concentration was about 1 mg/mL. An injection volume of 100 μL was used. The *M_w_* was calculated by the Zimm method of static light scattering based on the basic light scattering equation as follows:

$$\frac{Kc}{R_{\theta }}=\frac{1}{M_{w}}\left(1+\frac{16\pi^{2}<S^{2}{>}_{z}}{3\lambda^{2}}\sin^{2}(\frac{\theta }{2})\right)+2A_{2}c+\ldots ,$$

where *K* is an optical constant equal to [4*π*^2^*n*^2^(*dn*/*dc*)^2^]/(*N_A_λ*^4^); *c*, the polysaccharide concentration in g/mL; *R_θ_*, the Rayleigh ratio; *M_w_*, the weight average molecular mass; <*S^2^*>*_z_*^1/2^, the radius of gyration; *λ*, the wavelength; *n*, the refractive index of the solvent (0.9% NaCl aqueous solution); *dn*/*dc*, the refractive index increment of BG in 0.9% NaCl aqueous solution; *N_A_*, Avogadro’s number; *A*_2_, the second virial coefficient. The *dn/dc* value of BG was selected as 0.148 mL/g according to previous studies [31].

### 3.5. Determination of Intrinsic Viscosities

The intrinsic viscosities ([η]) of BG, BGD1 and BGD2 were measured by an Ubbelohde viscosity method reported previously with some modifications [32]. Briefly, the samples were dissolved in pure water, and then the solutions were taken into an Ubbelohde capillary viscometer at 25 °C for 15 min in a constant temperature bath. The kinetic energy correlation was assumed to be negligible, and the Huggins and Kraemer equations were used to estimate the value of [η] as follows,

$$\eta_{\mathrm{sp}}/c=\left[\eta \right]+k{\left[\eta \right]}^{2}c$$

$$(\ln\eta_{r})/c=\left[\eta \right]-\beta {\left[\eta \right]}^{2}c,$$

where *k* and β are constants for a given polymer under certain conditions in a given solvent; η_sp_/*c* is the reduced specific viscosity, and (ln η_r_)/*c* is the inherent viscosity; *c* is the polymer concentration.

### 3.6. In Vitro Binding Properties of Qingke β-Glucans with Different Molecular Weights

#### 3.6.1. Determination of Fat-Binding Capacity

The fa- binding capacities of Qingke β-glucans with different molecular weights (BG, BGD1, and BGD2) were measured according to a previously reported method with some modifications [33]. Briefly, 20 mg of each β-glucan was dissolved in 2.0 mL of pure water. Then 1 mL of peanut oil was added, and the mixture was incubated at 37 °C for 2 h with continuous shaking. After centrifugation (10,000× *g* for 20 min), the supernatant (considered as unbound oil) was carefully removed. The bound oil of β-glucans was extracted with petroleum ether for four times. Afterwards, the combined extracts were dried at 40 °C under a vacuum. The fat-binding capacity of Qingke β-glucans was expressed as gram of binding fat per gram of Qingke β-glucans (g/g). Cellulose was used as a positive control, and the pure water was used as a substrate blank. The test was conducted in triplicate.

#### 3.6.2. Determination of Cholesterol-Binding Capacity

The cholesterol-binding capacities of Qingke β-glucans with different molecular weights were measured according to a previously reported method with minor modifications [33]. Briefly, 20 mL of cholesterol micellar solution were prepared by sonication at 480 W for 1 h (PL-S80TX, Kangshijie company, Guangdong, China), composed of 10 mM sodium taurocholate, 5 mM cholesterol, 5 mM oleic acid, 132 mM sodium chloride, and 15 mM sodium phosphate buffer (pH 7.4). Then, 10 mg of Qingke β-glucans (BG, BGD1, and BGD2, respectively) were added into 3 mL of micellar solution, and the mixture was incubated at 37 °C for 2 h with continuous shaking. After centrifugation (13,000× *g*, 1 h), the supernatant was collected for the determination of cholesterol. The content of cholesterol in the supernatant was measured by the free cholesterol kit. In brief, 20 µL of supernatant were mixed with 980 µL of free cholesterol working solution. After incubation at 40 °C for 5 min, the absorbance was measured at 500 nm using a Varioskan Flash Multimode Reader (ThermoFisher, Waltham, MA, USA). Meanwhile, the content of binding cholesterol was calculated as the amount of cholesterol in the supernatant of the substrate blank subtracted from the amount in the supernatant of the sample. The cholesterol-binding capacity of Qingke β-glucans was expressed as milligrams of binding cholesterol per gram of Qingke β-glucans (mg/g). Cellulose was used as a positive control, and pure water was used as the substrate blank control.

#### 3.6.3. Determination of Bile Acid-Binding Capacity

The bile acid-binding capacities of Qingke β-glucans with different molecular weights were measured according to a previously reported method with minor modifications [20]. Briefly, the bile acid mixture was prepared with sodium cholate, sodium deoxycholate, sodium glycocholate, and sodium taurocholate with proportions as 35%, 35%, 15%, and 15% (*w*/*w*) in 50 mM phosphate buffer (pH 6.9), respectively. Each sample (20 mg) was digested with 1 mL of 0.01 M HCl in a shaking water bath at 37 °C for 1 h, which simulated gastric digestion. Then, to each sample, 4 mL of 1.4 µM bile acid mixture and 5 mL of porcine pancreatin (10 U/mL, to provide amylase, protease, and lipase for digestion) were added, and incubated at 37 °C for 1 h with continuous shaking. After centrifugation (13,000× *g*, 1 h), the supernatant was collected for the determination of unbound bile acid. The binding bile acid was calculated as the amount of bile acid in the supernatant of the substrate blank subtracted from the amount in the supernatant of the sample. The content of bile acid was measured by the furfural–sulfuric acid method [34]. The bile acid-binding capacity of Qingke β-glucans was expressed as a percent of blank control (%), and the cholestyramine and cellulose were used as positive and negative controls, respectively.

### 3.7. In Vitro Inhibitory Effects of β-Glucans with Different Molecular Weights on the α-Amylase and Pancreatic Lipase

The α-amylase inhibitory effects of Qingke β-glucans with different molecular weights was measured according to the procedure described previously with slight modifications [35]. In brief, 100 µL of each sample at different concentrations (1.0, 2.5, 5.0, and 10.0 mg/mL, respectively) was mixed with 100 µL of α-amylase solution (30 U/mL), and incubated at 37 °C for 30 min with continuous shaking. Then, 200 µL of soluble starch (0.5%, *w*/*v*) was added into the mixture, and incubated at 37 °C for 10 min. Subsequently, 1.6 mL of 3,5-dinitrosalicylic acid (DNS) reagent was added into the mixture, and incubated at a boiling water bath for 5 min. Finally, the absorbance of the mixture was measured at 540 nm. The α-amylase inhibitory activity was calculated as follows:

$$\mathrm{Inhibition}\left(\%\right)=\left(1-\frac{A_{\mathrm{sample}}-A_{\mathrm{control}}}{A_{\mathrm{blank}}-A_{\mathrm{control}}}\right)\times 100\%,$$

where A_sample_ is the absorbance of the mixture of sample, starch solution, α-amylase, and DNS reagent; A_control_ is the absorbance of the mixture of pure water (instead of sample), starch solution, and DNS reagent; A_blank_ is the absorbance of the mixture of pure water (instead of sample), starch solution, α-amylase, and DNS reagent.

Furthermore, the inhibition of pancreatic lipase activity was measured according to previously reported methods with minor modifications [24,33]. Briefly, 100 µL of each Qingke β-glucan at the concentrations of 0.25, 0.5, 1.0, 2.5, 5.0 and 10.0 mg/mL was mixed with 200 µL of Tris buffer (50 mM, pH 7.4), and 100 µL of pancreatic lipase solution (5 mg/mL), and the mixture was incubated at 37 °C for 10 min with continuous shaking. Then, 100 µL of *p*-nitrophenyl acetate (2 mM) were added and incubated at 37 °C for 15 min. Finally, the absorbance was measured at 405 nm, and a commercial capsule of orlistat was used as a positive control. The inhibition of pancreatic lipase was calculated according to the following equation:

$$\mathrm{Inhibition}\left(\%\right)=\left(1-\frac{A_{\mathrm{sample}}-A_{\mathrm{blank}}}{A_{\mathrm{control}}}\right)\times 100\%,$$

where A_sample_ is the absorbance of the mixture of sample, Tris buffer, pancreatic lipase, and *p*-nitrophenyl acetate solution; A_blank_ is the absorbance of the mixture of sample, Tris buffer, pure water (instead of pancreatic lipase solution), and *p*-nitrophenyl acetate solution; A_control_ is the absorbance of the mixture of pure water (instead of sample), Tris buffer, pancreatic lipase, and *p*-nitrophenyl acetate solution.

### 3.8. In Vitro Anti-Inflammatory Activity of Qingke β-Glucans with Different Molecular Weights

#### 3.8.1. Cell Culture

RAW 264.7 cells were purchased from Stem Cell Bank, Chinese Academy of Sciences, and cultured with DMEM solution (10% fetal bovine serum, 100 U/mL penicillin, and 100 μg/mL streptomycin) with 5% CO_2_ at 37 °C.

#### 3.8.2. Cell Viability

Cell viabilities of Qingke β-glucans with different molecular weights (BG, BGD1, and BGD2) were determined by MTT colorimetric method [36]. Briefly, RAW 264.7 cells were seeded into 96-well plates at a density of 1 × 10^4^ cells per well and incubated for 12 h. Cells were treated with different concentrations (0.25, 0.5, 1.0, and 2.0 mg/mL) of BG, BGD1, and BGD2 in the presence of LPS (10 μg/mL) for 24 h, respectively. An equal volume of culture medium was used as the blank control. Then, 10 μL of MTT working solution (5 mg/mL) were added and incubated for 4 h at 37 °C. After adding 100 μL of DMSO to each well, the absorbance was measured at 570 nm using a microplate reader (SpectraMax M5, Molecular Devices, Sunnyvale, CA, USA).

#### 3.8.3. NO Production

RAW 264.7 cells (1 × 10^4^/well) were seeded in 96-well microplates overnight, and then incubated with serial concentrations (0.2, 0.4, 0.6, 0.8, and 1.0 mg/mL) of BG, BGD1, and BGD2 in the presence of LPS (10 μg/mL) for 24 h. The culture medium was used as the blank control. Subsequently, 100 μL of supernatants were mixed with an equal volume of modified Griess reagent at room temperature for 15 min. The absorbance was measured at 540 nm with a microplate reader (SpectraMax M5, Molecular Devices). The nitric oxide (NO) production was expressed as the ratio of absorbance values between treatment groups.

### 3.9. In Vitro Anti-Cancer Activity of Qingke β-Glucans with Different Molecular Weights

The in vitro anti-cancer activities of Qingke β-glucans with different molecular weights (BG, BGD1, and BGD2) were measured by the MTT colorimetric method [36]. Human colon cancer cells (HCT116), human breast cancer cells (MDA-MB-231), and human lung cancer cells (A549) were obtained from the American Type Culture Collection (ATCC, Manassas, VA, USA) and cultured in DMEM solution with 5% CO_2_ at 37 °C, respectively. Each cell suspension was seeded in 96-well plates and incubated for 24 h at a concentration of 8 × 10^5^ cell/mL. One hundred microliters of BG, BGD1, and BGD2 solutions (0.125, 0.25, 0.5, 1.0, and 2.0 mg/mL) were added into the wells in turn. After incubation for 24 h, 10 μL of MTT (5 mg/mL) were added and incubated for 4 h at 37 °C. Then, culture media were removed, and 100 μL of DMSO were added to each well. Absorbance was measured at 570 nm using a microplate reader (SpectraMax M5, Molecular Devices, Sunnyvale, CA, USA), and the anti-cancer activity was expressed as the viability of cell growth.

### 3.10. Statistical Analysis

All experiments were conducted in triplicate, and data were expressed as mean ± standard deviation. Statistical analysis was performed using Origin 9.0 software (OriginLab Corporation, Northampton, MA, USA). Statistical significance tests were carried out by one-way analysis of variance (ANOVA) and Student’s *t*-test. Values of *p* < 0.05 were considered statistically significant.

## 4. Conclusions

In the present study, the correlations of molecular weights of Qingke β-glucans to their in vitro binding properties, inhibitory activities on digestive enzymes, anti-inflammatory activities, and anti-cancer activities were systematically investigated. Results showed that the in vitro binding properties and the inhibitory activities on digestive enzymes of Qingke β-glucans decreased as the molecular weights decreased. However, the anti-inflammatory activities of Qingke β-glucans increased as the molecular weights decreased. Furthermore, Qingke β-glucans exhibited selective anti-cancer activity in vitro. Positive and negative correlations of molecular weights of Qingke β-glucans to their in vitro growth inhibitory effects against A549 cells and MDA-MB-231 cells were observed, respectively. However, further in vitro and in vivo studies are required to confirm and clarify the possible mechanisms of these bioactivities. Results suggested that molecular weights of Qingke β-glucans significantly affected their bioactivities, which was beneficial for a better understanding of their structure–function relationships. Moreover, results showed that Qingke β-glucans could be further explored as functional/healthy food ingredients for industrial applications due to their multiple health benefits.

## Figures and Tables

**Table 1:** Molecular weights (*M_w_*), polydispersities (*M_w_*/*M_n_*), intrinsic viscosities ([η]), purities, and proteins of Qingke β-glucans.

Sample	Molecular Weight (g/mol)		*M_w_*/*M_n_*	Purity (%)	Protein (%)	[η] (dL/g)
	*M_n_* × 10^5^ (Error)	*M_w_* × 10^5^ (Error)				
**BG**	1.236 (±0.69%)	1.962 (±0.54%)	1.59	93.8	2.15	1.86
**BGD1**	0.456 (±2.27%)	0.638 (±2.35%)	1.40	93.1	0.72	0.98
**BGD2**	0.245 (±3.14%)	0.358 (±2.65%)	1.46	93.3	0.49	0.58

**BG**, Qingke β-glucan; **BGD1**, Qingke β-glucan with acid hydrolysis for 10 min; **BGD2**, Qingke β-glucan with acid hydrolysis for 20 min.

**Table 2:** The fat-binding, cholesterol-binding, and bile acid-binding capacities of Qingke β-glucans.

Sample	Fat Binding (g/g)	Cholesterol Binding (mg/g)	Bile Acid Binding (%)
**BG**	2.23 ± 0.03 ^a^	40.37 ± 0.43 ^a^	26.49 ± 0.18 ^b^
**BGD1**	1.77 ± 0.02 ^b^	22.68 ± 0.31 ^b^	23.39 ± 0.22 ^c^
**BGD2**	1.39 ± 0.02 ^c^	10.56 ± 0.28 ^d^	11.54 ± 0.25 ^d^
**PC**	0.89 ± 0.01 ^d^	18.59 ± 0.18 ^c^	41.53 ± 0.15 ^a^
**NC**	N/A	N/A	3.81 ± 0.21 ^e^

**BG**, Qingke β-glucan; **BGD1**, Qingke β-glucan with acid hydrolysis for 10 min; **BGD2**, Qingke β-glucan with acid hydrolysis for 20 min; **PC**, positive control; **NC**, negative control; **N/A**, not available; cellulose was used as a positive control in fat-binding and cholesterol-binding assay, respectively, and cholestyramine and cellulose were used as positive and negative controls in bile acid binding assay; values represent mean ± standard deviation, and different letters (a–e) in the same column indicate significant differences (*p* < 0.05).
